# Heat Shock Tolerance in *Deschampsia antarctica* Desv. Cultivated *in vitro* Is Mediated by Enzymatic and Non-enzymatic Antioxidants

**DOI:** 10.3389/fpls.2021.635491

**Published:** 2021-02-23

**Authors:** Rodrigo Cortés-Antiquera, Marisol Pizarro, Rodrigo A. Contreras, Hans Köhler, Gustavo E. Zúñiga

**Affiliations:** ^1^Departamento de Biologia, Facultad de Química y Biología, Universidad de Santago de Chile, Santiago, Chile; ^2^Centro para el Desarrollo de la Nanociencia y la Nanotecnología (CEDENNA), Santiago, Chile

**Keywords:** Antarctica, climate change, oxidative stress–related enzymes, peroxidases, photosynthesis

## Abstract

*Deschampsia antarctica* Desv, is the most successful colonizing species of a cold continent. In recent years due to climate change, the frequency of heat waves has increased in Antarctica, registering anomalous high temperatures during the summer of 2020. However, the populations of *D. antarctica* are responding positively to these events, increasing in number and size throughout the Antarctic Peninsula. In this work, the physiological and biochemical responses of *D. antarctica* plants grown *in vitro* (15 ± 1°C) and plants subjected to two heat shock treatments (23 and 35°C) were evaluated. The results obtained show that *D. antarctica* grown *in vitro* is capable of tolerating heat shock treatments; without showing visible damage to its morphology, or changes in its oxidative state and photosynthetic performance. These tolerance responses are primarily mediated by the efficient role of enzymatic and non-enzymatic antioxidant systems that maintain redox balance at higher temperatures. It is postulated that these mechanisms also operate in plants under natural conditions when exposed to environmental stresses.

## Introduction

Antarctica is considered the most severe ecosystem in the world (Robinson et al., 2003). The temperature experiences daily fluctuations of −10°C to +15°C during summer (Convey, 2013). These extreme climatic conditions explain the scarce plant biodiversity that exists in Antarctica (Convey, 2006), it has allowed that only two vascular plants have naturally colonized the Maritime Antarctica, *Colobanthus quitensis* (Kunth) Bartl caryophyllaceae and *Deschampsia antarctica* Desv poaceae.

Both Antarctic plants differ in their reproductive capacities (Gielwanoska and Szczuka, 2005), being *D. antarctica* the most successful colonizer of the Antarctic continent, compared to *C. quitensis* (Cavieres et al., 2016). *D. antarctica* is the most abundant and widely distributed throughout maritime Antarctica (Smith, 2003; Sáez et al., 2018). The plasticity of *D. antarctica* is a key to response to fluctuating environmental conditions (Gielwanoska et al., 2005). Anatomical modifications of the leaf anatomy present similitudes with Kranz anatomy associated with carbon metabolism, C4, furthermore presenting characteristics [R] more xeromorphic and ultrastructure differences in chloroplasts and mitochondria (Romero et al., 1999; Mantovani and Vieira, 2000; Gielwanoska and Szczuka, 2005), that improves the water-use efficiency (Montiel et al., 1999) and photosynthetic efficiency (Xiong et al., 1999; Sáez et al., 2017, 2018).

The effects of climate change are evident throughout the Antarctic Peninsula, recording the fastest atmospheric warming in the last 50 years (Turner and Overland, 2009). In this period, the average temperature in Antarctica increased by 3.7°C, a value much higher than the rest of the world (Turner et al., 2013). During the summer of 2020 in Antarctica, heatwaves were recorded at the Marambio base on Seymour Island, on the east side of the Antarctic peninsula, exceeding 20°C (Robinson et al., 2020). These warmer temperatures in Antarctica have produced dramatic changes on the continent: longer growing seasons with high temperatures, the retreat of glaciers, an increment of ice-free areas, and increased frequency of rainfall (Cavieres et al., 2016; Sáez et al., 2018). Warmer temperatures do not affect the landscapes, but could also affect the biota of the continent, changing the distribution and abundance of populations. In nature, any abrupt and short temperature increase of 5°C or more above the optimal growth temperature can be considered as a heat shock (Bita and Gerats, 2013). If the time of exposure to thermal shock is prolonged, it can irreversibly damage the stability of proteins, membranes, cytoskeletal structures, and RNA, affecting the growth and development of plants (Driendoks et al., 2015).

Although these negative effects of global warming on plants, populations of *D. antarctica* are increasing in number and size along the Antarctic Peninsula: King George Island (Torres-Mellado et al., 2011), Byers Peninsula, Livingston Island (Vera et al., 2013), Isla Robert (Torres-Mellado et al., 2011) and even in Argentine islands (Fowbert and Smith, 1994). The distribution of *D. antarctica* is highly influenced by climatic conditions (Parnikoza et al., 2011), studies carried out in ground conditions show that at warmer temperatures (13°C and 19°C), *D. antarctica* obtains the maximum CO_2_ assimilation rate, improving its photosynthetic performance and growth (Edwards and Smith, 1988; Sáez et al., 2017, 2018). This may explain the reproductive success of *D. antarctica* even in warmer conditions. It has been suggested that in the future, high temperatures could induce morphological and anatomical changes in Antarctic plants to maximize light capture and growth (Cavieres et al., 2016). Due to their successful adaptation to climate change and its rapid spread, *D. antarctica* has been suggested as an ecological marker for global warming and a valuable resource for understanding stress tolerance (Lee et al., 2012).

Plants have developed different mechanisms to tolerate heat shock: maintaining the stability of cell membranes, ROS uptake, antioxidant biosynthesis, accumulation of compatible osmolytes, induction of intracellular signaling cascades and the action of molecular chaperones (Wahid et al., 2007; Bita and Gerats, 2013). Several studies on *D. antarctica* have shown the effective role of antioxidants to respond to various types of abiotic stress such as osmotic stress (Zamora et al., 2010) and UV-B radiation stress (Sequeida et al., 2012; Köhler et al., 2017). While in heat shock events, the accumulation of heat shock protein 70 (Hsp70) in leaves has been described (Reyes et al., 2003) and the crucial high affinity of Rubisco for CO_2_ to optimize carbon assimilation (Sáez et al., 2018). Studies of *in situ* heating have provided important information on the response to thermal shock in *D. antarctica*. Working with plants grown *in vitro* allows to control environmental variables and expose *D. antarctica* to unusual conditions of heat shock, which contribute to clearly analyze the responses generated. We hypothesize that *D. antarctica* has efficient antioxidant protection mechanisms in response to heat shock treatment, being able to maintain normal physiology, photosynthetic performance, and redox state balance.

The objective of this work was to evaluate the capacity of *D. antarctica* cultivated *in vitro* to tolerate heat shock treatment, analyzing the effect of temperature on the redox balance, photosynthetic performance, and various enzymatic like SOD, POD, CAT, APX, and non-enzymatic antioxidant activities.

## Materials and Methods

### Plant Material

Plants of *D. antarctica* plants were grown *in vitro* at the Plant Physiology and Biotechnology Laboratory (Universidad Santiago de Chile). The plant material was micropropagated using Murashige and Skoog basal medium, supplemented with 20 g/L of sucrose, 5 mM myo-inositol, 2 mg/L naphthaleneacetic acid (NAA), 0.8 mg/L benzyl amino purine (BAP) and 4 g/L of activated carbon. The pH of the medium was adjusted to 5.6 and 6 g/L of agar-agar was added as a gelling agent. The culture medium was sterilized in an autoclave at 121°C and a pressure of 15 pounds. The *in vitro* plants were kept under controlled conditions at 15 ± 1°C and 16:8 photoperiods (16 h of light and 8 h of night) in a culture chamber at 70% of relative humidity. All *in vitro* plants used in the experiments were two-months old.

### Lethal Temperature 50

The lethal temperature (LT_50_) is defined as the temperature necessary to kill 50% of the leaf tissue and was determined in *D. antarctica* grown *in vitro*. 100 mg of plant tissue were resuspended in 5 mL of deionized water in a Falcon tube and kept in a block heater (Thermo Scientific, Spain) for 3 h in a temperature range of 15–100°C. After This period, the plants were kept at room temperature for 10 min, and the maximum photosynthetic efficiency of photosystem II (PSII) (Fv/Fm) of the plant was determined as a survival parameter. The degree of damage caused by heat treatments was calculated considering 100% damage at a temperature of 100°C. The data obtained in the treatments were used in the determination of the LT_50_ in *D. antarctica* plants grown *in vitro*.

### Heat Shock Treatment

Two heat shock treatments were used at 23 and 35°C, (8 and 20°C above the optimum growth temperature under laboratory conditions, respectively, for *D. antarctica* plants grown *in vitro*. This selection temperature was based on field temperature data recorded by our group in the South Shetlands Islands over the past 5 years (data not yet published). The plants were removed from the culture chamber at 15°C and exposed to the thermal shock treatments for 2 h (8–10 am) using a heating bath (Memmert, Germany) for a period of 7 days. The relative humidity inside the culture vessel was over 90%. The analyzes for both treated and control plants were performed on days 1, 2, 3, and 7. The aerial growth of the seedlings was evaluated immediately after the respective samplings, using ImageJ software, the samples were frozen in liquid nitrogen and stored at −80°C for later analysis.

### Oxidative Stress Parameters

ROS’s total content was measured by fluorometric quantification of dichlorodihydrofluorescein-diacetate (DCDHF-DA) oxidized by the presence of ROS. 50 mg of fresh plant tissue were incubated in 1 mL of 10 mM DCDHF-DA in Tris–HCl buffer (50 mM, pH 8.0) for 1 h at 37°C in dark conditions. Then, the tissue was washed twice with a 50 mM EDTA solution to remove the remains of the incubation solution, and the tissue was ground under liquid nitrogen to a powder and extracted into 1 ml of Tris–HCl buffer (50 mM, pH 8.0) and centrifuged at 10,000 rpm for 10 min. The obtained supernatant was used to determine the fluorescence, using an excitation wavelength of 488 nm and an emission wavelength of 535 nm in a microplate reader spectrophotometer (TECAN, Infinite2000pro, Austria). The ROS content was determined using an oxidized DCF-DA calibration curve (Ross et al., 2008).

Lipid peroxidation was estimated by measuring the concentration of malondialdehyde (MDA) by the thiobarbituric acid reactive substances (TBARS) assay. 50 mg of fresh plant tissue was ground in liquid nitrogen to a powder and suspended in 1 ml of 1% trichloroacetic acid (TCA). Then the homogenate was centrifuged at 13,000 rpm for 5 min. 250 μL of the supernatant was mixed with 1 mL of 0.5% thiobarbituric acid (TBA) in 20% TCA. The mixture was boiled for 30 min and cooled to room temperature. The MDA formed was quantified at 532 nm and 600 nm in a microplate reader spectrophotometer (TECAN, Infinite2000pro, Austria), using a molar extinction coefficient of 155 mM-1 cm-1 (Ederli et al., 2004).

### Photosynthetic Efficiency

Chlorophyll fluorescence was analyzed using the portable fluorometer PEA (Hansatech, England). The leaves were held in commercial leaf clips (Hansatech, England) that allow keeping the samples in the dark before the measurement (30 min) (Stirbet and Govindjee, 2011).

From the fluorescence induction signal from 10 μs to 3 s the instrument determines initial (Fo) and maximum (Fm) fluorescence and the variable fluorescence (Fv) at specified time intervals.

Furthermore, it calculates specific parameters such as the maximum efficiency of PSII (Fv/Fm), maximum quantum yield PSII (Φ PSII), and electron transport rate (ETR) (Stirbet and Govindjee, 2011).

### Photosynthetic Pigments Content

The total content of chlorophyll and carotenoids was determined by the method of Wellburn and Lichtenthaler (1984). 100 mg of fresh plant tissue was used and macerated with liquid nitrogen in 1 mL of cold 80% acetone. The mixture was centrifuged at 13,000 rpm for 10 min and the supernatant was recovered. Each sample was diluted 1:10 and absorbance was measured at 470 nm, 649 nm, and 665 nm in a microplate reader spectrophotometer (TECAN, Infinite2000pro, Austria). Chlorophyll concentration was determined using Eqs. (1) and (2), and the ratio between chlorophyll was determined using Eq. (3). Carotenoids concentration was determined using Eq. (4)

(1) $$C\,h\,l\,o\,r\,o\,p\,h\,y\,l\,l\,a\,\left(\mu \,g/\text{mL}\right)=\left(12,25*A_{665}-2,79*A_{649}\right)$$

(2) $$C\,h\,l\,o\,r\,o\,p\,h\,y\,l\,l\,b\,\left(\mu \,g/\text{mL}\right)=\left(21,2*A_{649}-2,79*A_{665}\right)$$

(3) $$C\,h\,l\,o\,r\,o\,p\,h\,y\,l\,l\,r\,a\,t\,i\,o=\frac{\left[C\,h\,l-a\right]}{\left[C\,h\,l-b\right]}$$

(4) $$C\,a\,r\,o\,t\,e\,n\,o\,i\,d\,s\,\left(\mu \,g/\text{mL}\right)=\frac{1000*A_{470}-1,82*\left[C\,h\,l-a\right]-85,02*\left[C\,h\,l-b\right]}{198}$$

### Enzyme Extraction and Quantification

The protein extraction was conducted using 100 mg of fresh plant tissue and was ground to a powder and suspended in 1 mL of sodium phosphate buffer (50 mM, pH 7.5), and then the mixture was centrifuged at 10,000 rpm for 10 min at 4°C. The supernatant was recovered, and the total protein content was determined according to Bradford method (Bradford, 1976). The absorbance was measured at 595 nm on a UV-Vis spectrophotometer (Agilent 8453, Santa Clara, CA, United States). The total protein content was calculated using Bovine Serum Albumin (BSA) as a calibration standard (Bradford, 1976).

### Antioxidant Enzymatic Activity

Superoxide dismutase activity (SOD, EC 1.15.1) was determined by using the photoinhibition of nitro-blue tetrazolium assay (NBT) (Beauchamp and Fridovich, 1971). A reaction mixture was prepared using 600 μL of sodium phosphate buffer (50 mM, pH 7.5), 10 μL of 10 mM EDTA, 100 μL of 130 mM methionine, 10 μL of 2 mM riboflavin, 200 μL of 3 mM of nitroblue tetrazolium (NBT) in 70% dimethylformamide (DMF) and 100 μL of protein extract. The mixture was incubated under white light for 15 min at room temperature (a blank kept in the dark). Absorbance at 560 nm was determined where one enzymatic unit (EU) was considered as the capacity to inhibit 50% of photochemical reduction of NBT (Köhler et al., 2017).

Total peroxidase class III activity (POD, EC 1.11.1.7) was determined by tetrahydro-guaiacol (THG) formation using peroxide and guaiacol as substrates (Pinhero et al., 1997). A reaction mixture that contained 1 mL of sodium phosphate buffer (50 mM, pH 7.5), 10 μL of protein extract, 5 μL of 100 vol. hydrogen peroxide and 5 μL of guaiacol was prepared. Absorbance at 470 nm was recorded after a reaction time of 1 min indicating POD activity in terms of tetrahydroguaiacol (THG) formation. POD activity was calculated using molar extinction of THG, ε = 26.6 mM-1 cm-1 (Köhler et al., 2017).

Ascorbate peroxidase (APX, EC 1.11.1.11) was determined by ascorbate consumption for 60 s (Lima et al., 2002). A reaction mixture that contained 1 mL of sodium phosphate buffer (50 mM, pH 7.5), 20 μL of protein extract, 5 μL of 100 vol. hydrogen peroxide and 40 μL of 10 mM sodium ascorbate was prepared. Absorbance at 290 nm was recorded after the reaction had proceeded for 1 min indicating APX activity in terms of ascorbate consumption. APX activity was calculated using molar extinction of ascorbate, ε = 2.8 mM-1 cm-1 (Köhler et al., 2017).

Catalase activity (CAT, EC 1.11.1.6) was determined by H_2_O_2_ consumption for 60 s (Lima et al., 2002). A reaction mixture that contained 900 μL of sodium phosphate buffer (50 mM, pH 7.5), 10 μL of protein extract and 3 μL of 100 vol. hydrogen peroxide was prepared. Absorbance at 240 nm was recorded after a reaction time of 1 min indicating CAT activity in terms of hydrogen peroxide consumption. CAT activity was calculated using molar extinction of hydrogen peroxide, ε = 39.4 mM-1 cm-1 (Köhler et al., 2017).

### Hydroalcoholic Extracts Preparation

Hydroalcoholic extracts were prepared using 100 mg fresh plant tissue and were ground to a powder and suspended in 1 mL ethanol (85% v/v) and sonicated at 50-60 Hz for 3 h at room temperature. Extracts were filtered in a 0.45 um pore filter (Millipore, Billerica, MA, United States) and used for non-enzymatic antioxidant assays (Contreras et al., 2015).

### Total Phenolic Content

The total phenolics content was determined by the modified Folin-Ciocalteu colorimetric method (Asami et al., 2003). A reaction mixture was prepared with 115 μL of deionized water, 20 μL of Folin-Ciocalteu reagent diluted (1:10), and 5 μL of hydroalcoholic extract. The mixture was incubated in the dark for 15 min at room temperature, then 60 μL of sodium carbonate (7% w / v) was added and incubated for 1 h at 35°C. The absorbance was measured at 740 nm in a microplate reader spectrophotometer (TECAN, Infinite2000pro, Austria). The total phenol content was expressed in gallic acid equivalents (GA) per gram of fresh weight (FW).

### Non-enzymatic Antioxidant Activity

Antioxidant activity of hydroalcoholic extracts was measured by the bleaching of 1,1-Difenil-2-Picril-Hidrazil (DPPH) cation radical (Brand-Williams et al., 1995). A reaction mixture with 100 μL DPPH 100 mM (A517 = 0,7 − 0,8) and 10 μL protein extract was prepared. Absorbance at 515 nm was measured for 10 min at 37°C (TECAN, Infinite2000pro, Austria). The antioxidant activity were expressed as DPPH consumption percentage (%), where DPPH 100 mM was used as a control reference (Naik et al., 2005).

### Statistical Analysis

All experiments were performed in triplicate, for statistically significant differences we used two-way ANOVA with multiple comparisons. Post statistical analysis were performed using Tukey’s post-test (*P* < 0.05).

## Results

### LT_50_ Determination

**FIGURE 1:**
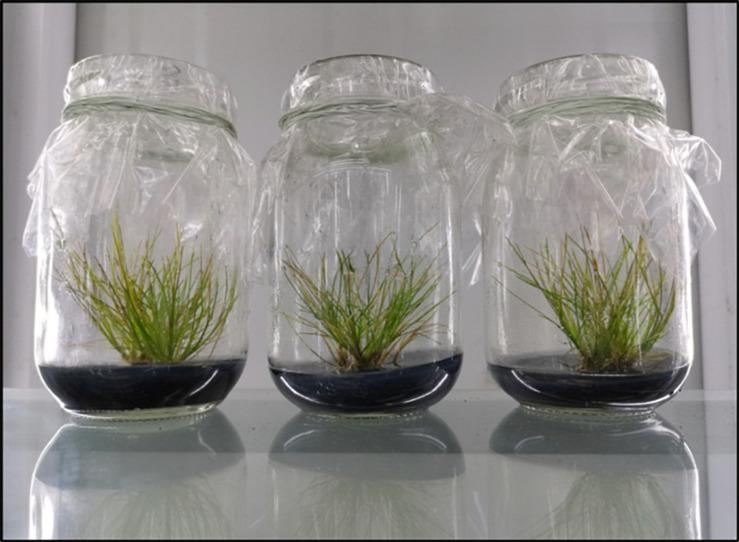
*In vitro* grown *D. antarctica* plants used in the experiments. Each culture vessel is considered a biological sample.

**FIGURE 2:**
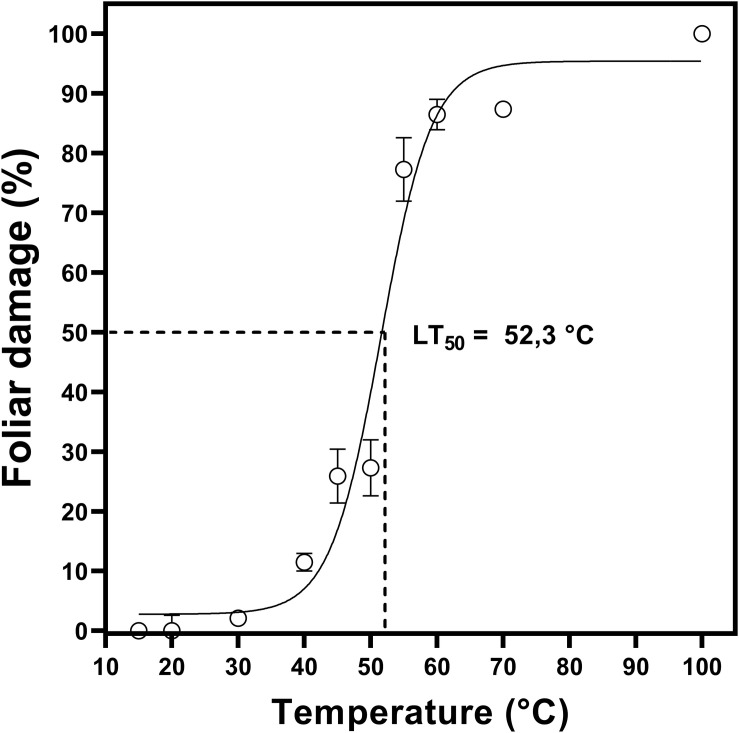
Sublethal temperature 50 (LT_50_) in *D. antarctica* cultivated *in vitro.* The photosynthetic parameter Fv/Fm was expressed like foliar damage *(%).* Each point represent means of 3 biological replicates (*N* = 3; ± standard error of the mean).

Two-month-old plants were used in the determination of LT_50_ (Figure 1). To determine heat tolerance, we calculated LT_50_ values based on the measurement of maximum PSII efficiency (Fv/Fm) in *D. antarctica* cultured *in vitro*. From this analysis, it was determined that the LT*50* in *D. antarctica* shoots, grown under laboratory conditions at 15°C, was 52°C (Figure 2).

### Heat Shock Effect on Physiology and Photosynthetic Efficiency

**FIGURE 3:**
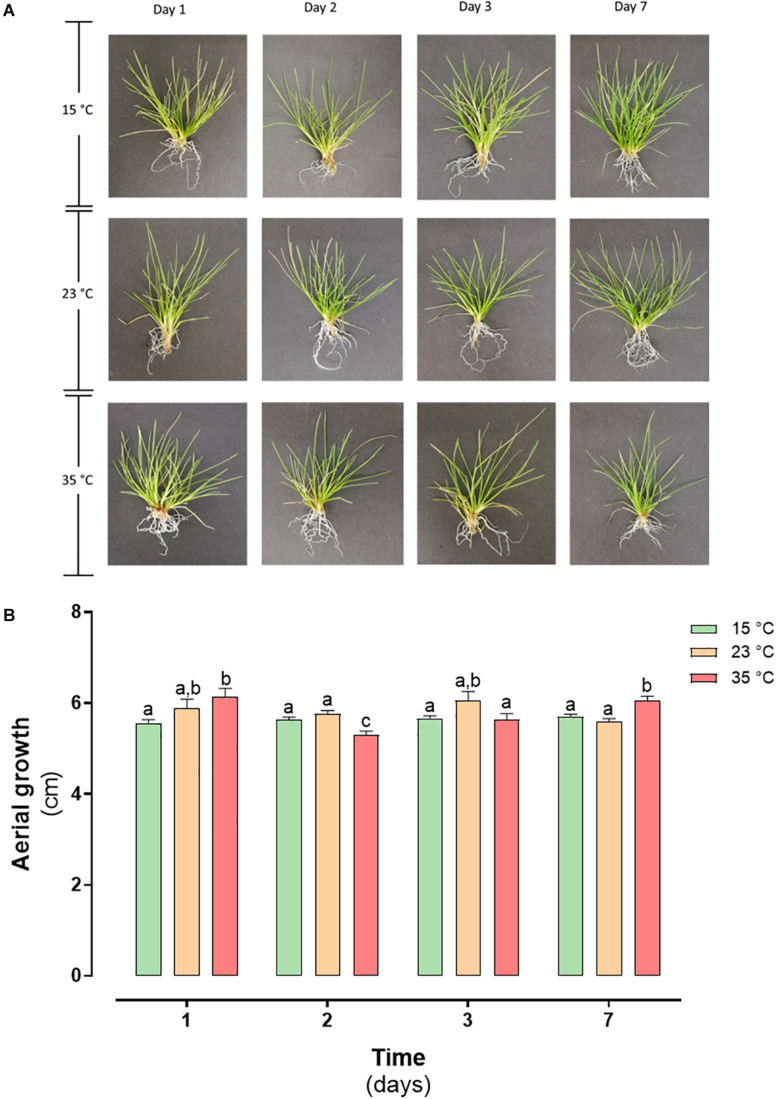
Heat shock effect in morphology and growth in *D. antarctica* control (15°C) and heat shock treatments (23°C and 35°C) cultivated *in vitro*. **(A)** Photographs of *D. antarctica* expose to heat shock treatments from day 1 at day 7 and **(B)** aerial growth. Each bar represent means of 3 biological replicates (*N* = 3; ± standard error of the mean). Significant differences between treatments are indicated by letters (*P* < 0.05).

From the normal growth temperature, the determined LT_50_ value, and the temperature data recorded in Antarctica, two thermal shock treatments were applied (23 and 35°C). The plants treated at both temperatures did not present morphological differences concerning the control (Figure 3A), maintaining their coloration and leaf shape. When evaluating the effect of the treatment on plant growth, no significant differences were observed in the plants’ aerial length (*P* < 0.05) (Figure 3B).

**FIGURE 4:**
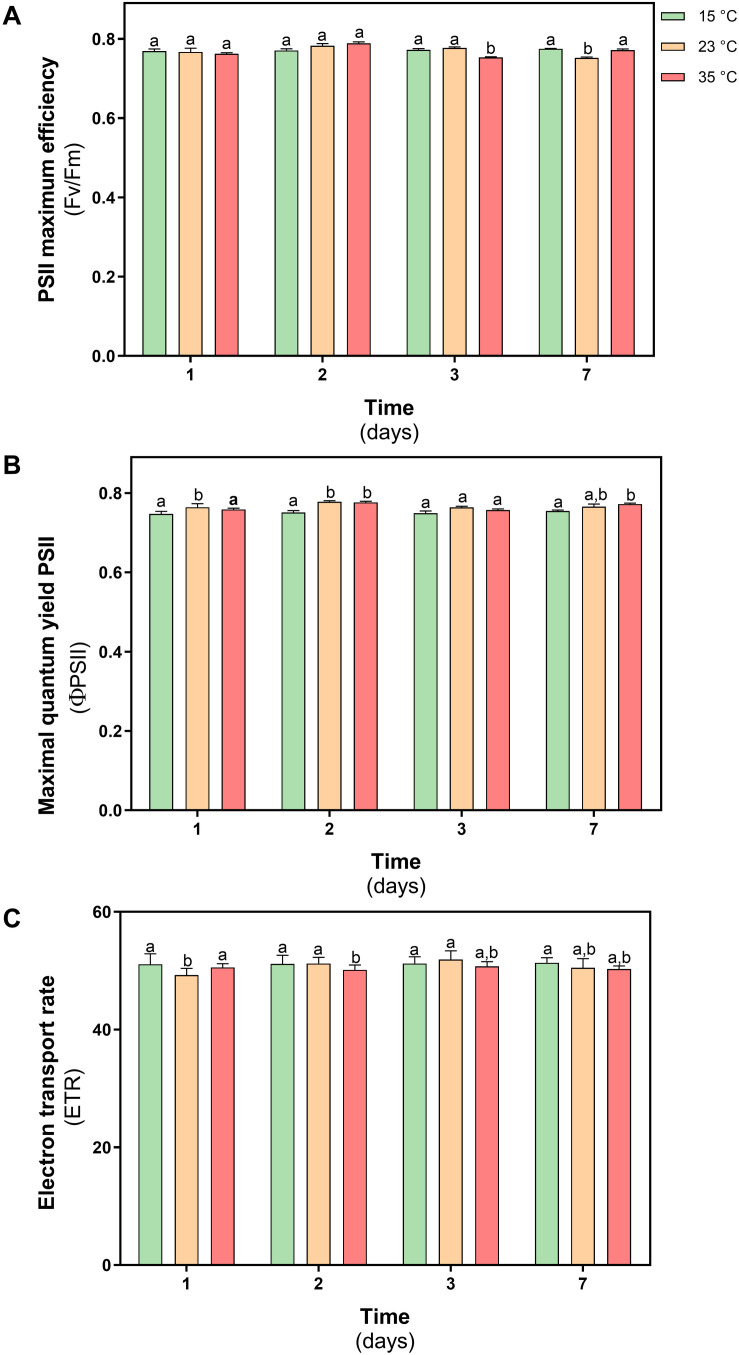
Photosynthetic efficiency parameters in *D. antarctica* control (15°C) and heat shock treatments (23°C and 35°C) cultivated *in vitro*. **(A)** PSII maximun efficiency (Fv/Fm), **(B)** maximal quantum yield PSII (ΦPSII), and **(C)** electron transport rate (ETR). Each bar represent means of 3 biological replicates (*N* = 3; ± standard error of the mean). Significant differences between treatments are indicated by letters (*P* < 0.05).

As shown in Figure 4, we examined three photosynthetic parameters: chlorophyll fluorescence (Fv/Fm), quantum yield of photosystem II (ΦPSII) and electron transport rate (ETR). The maximal photochemical efficiency (Fv/Fm) was in the range 0.70–0.80 at both temperatures of heat shock (Figure 4A). The effective photochemical efficiency (ΦPSII) and electron transport rate (ETR) did not show any significant changes in *D. antarctica* exposed to heat shock at 23 and 35°C compared with the control (Figures 4B,C, respectively). These results on *D. antarctica* are comparable with previous results reported in nonstressed plants.

**FIGURE 5:**
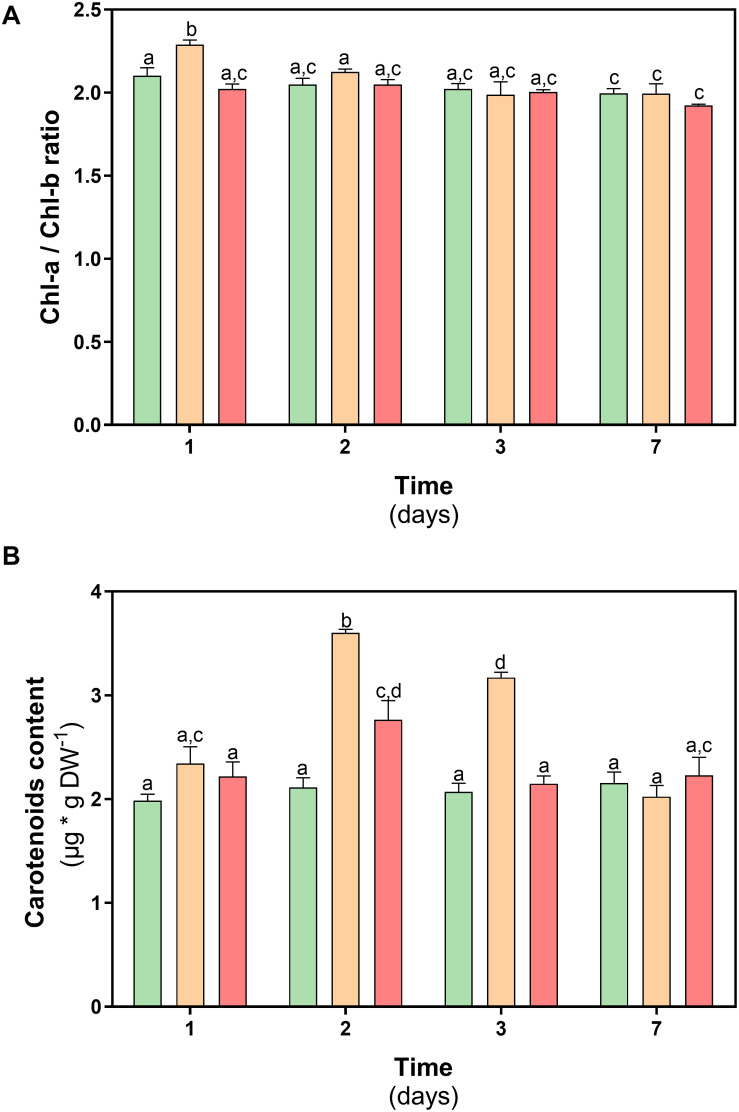
Photosynthetic pigments content in *D. antarctica* control (15°C) and heat shock treatments (23 and 35°C) cultivated *in vitro*. **(A)** chl-a/chl-b ratio and **(B)** carotenoids content. Each bar represent means of 3 biological replicates (*N* = 3; ± standard error of the mean). Significant differences between treatments are indicated by letters (*P* < 0.05).

With the purpose of complementing the previous results, the levels of photosynthetic pigments in *D. antarctica* were measured at both treatments. The results obtained show that the Chl-a / Chl-b ratio in plants subjected to thermal treatments remained within the range (1.8–2.5) described for nonstressed plants (Figure 5A). Additionally, the content of carotenoid pigments in *D. antarctica* showed a significant increase on day 2 of treatment (47% at 23°C and 21% at 35°C) but decreased at the end of the experiment reaching basal values (*P* < 0.05) (Figure 5B).

### Homeostasis Redox

**FIGURE 6:**
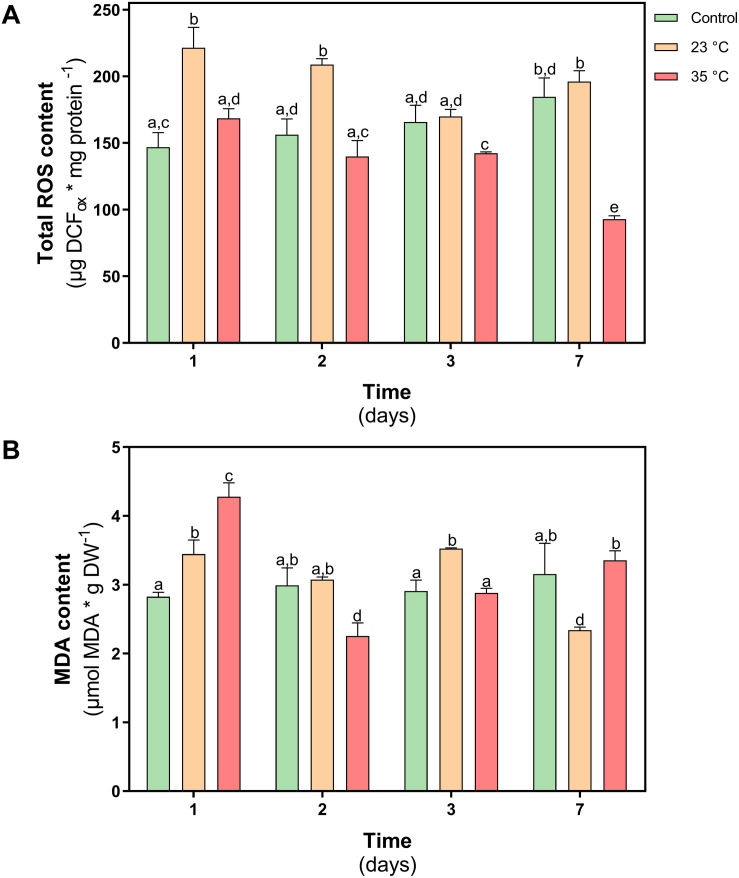
Oxidative stress parameters in *D. antarctica* control (15°C) and heat shock treatments (23°C and 35°C) cultivated *in vitro*. **(A)** Total ROS content and **(B)** membrane peroxidation (malondialdehyde content). Each bar represent means of 3 biological replicates (*N* = 3; ± standard error of the mean). Significant differences between treatments are indicated by letters (*P* < 0.05).

One of the effects caused by the increase in temperature in plants is to alter redox homeostasis. For this reason, the levels of reactive oxygen species (ROS) and the levels of membrane lipoperoxidation (MDA) was evaluated in *D. antarctica* plants subjected to both thermal shock treatments. In plants treated at 23°C, an increase in ROS levels was observed on day 1 of treatment (25%). However, on day 7 of treatment, no significant differences were observed with respect to the control plants. In the plants treated at 35°C, a progressive decrease in ROS levels was observed during the treatment, reaching a 50% decrease on day 7 of treatment (*P* < 0.05) (Figure 6A). The MDA content in both treatments showed an increase on day 1, reaching values of 19 and 47% at 23 and 35°C, respectively. Then the MDA levels decreased over time in both treatments, reaching the levels of the control plants (Figure 6B).

### Enzymatic Antioxidant System

**FIGURE 7:**
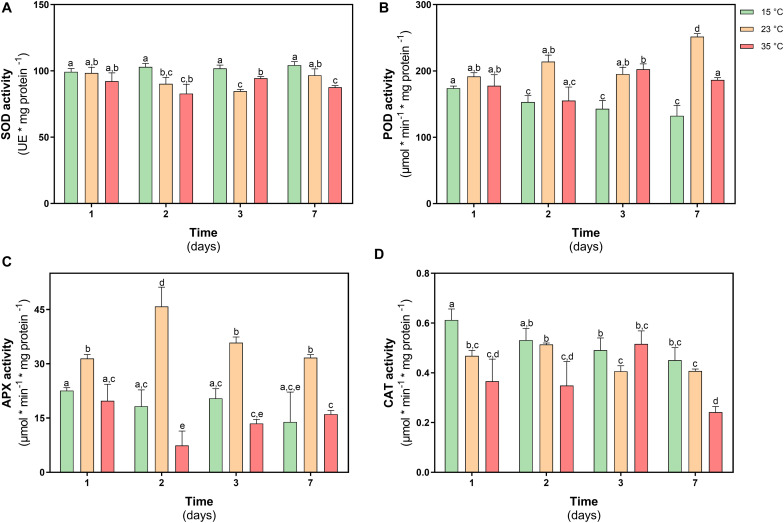
Antioxidant enzyme activity in *D. antarctica* control (15°C) and heat shock treatments (23°C and 35°C) cultivated *in vitro*. **(A)** superoxide dismutase (SOD) activity, **(B)** total peroxidases type III (POD) activity, **(C)** ascorbate peroxidase (APX) activity, and **(D)** catalase (CAT) activity. Each bar represent means of 3 biological replicates (*N* = 3; ± standard error of the mean). Significant differences between treatments are indicated by letters (*P* < 0.05).

The activity of superoxide dismutase (SOD) did not show differences in their activity at day 1, while through time only at 35°C showed a decrease of 13% at day 7 respect to control and under 23°C treatment SOD had similar levels of activity (Figure 7A).

The activity of type III peroxidases in *D. antarctica* (POD), showed an increase in their activity of 38% on day 7 in plants treated at 23°C, while in plants treated at 35°C an increase was only observed on day 3 treatment equivalent to 8% (Figure 7B). The activity of the APX enzyme only showed changes in the treatment at 23°C, increasing 33 and 60% on days 1 and 2 of treatment, respectively, with respect to the control plants (Figure 7C). The activity of the CAT enzyme showed a more erratic behavior during the treatment. This activity showed a decrease on days 1 and 2 with respect to the control plants (18 and 35%, respectively) (Figure 7D).

### Non-enzymatic Antioxidant System

**FIGURE 8:**
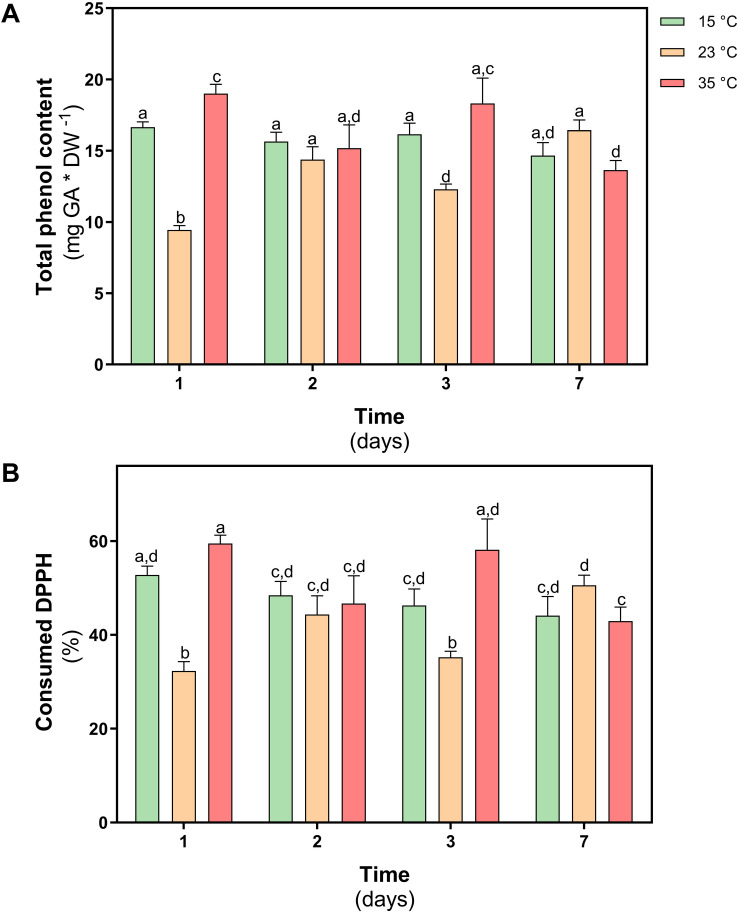
Non-enzymatic antioxidant activity in *D. antarctica* control (15°C) and heat shock treatments (23 and 35°C) cultivated *in vitro*. **(A)** total phenol content and **(B)** the consumption DPPH radical. Each bar represent means of 3 biological replicates (*N* = 3; ± standard error of the mean). Significant differences between treatments are indicated by letters (*P* < 0.05).

The content of total phenolic compounds was determined in both treatments. In *D. antarctica* plants subjected to 35°C, an increase of 10% was observed on day 1 with respect to the control, however, in plants treated at 23°C, a 45% decrease was observed on day 1. At day 7 of treatment, the values of the content of total phenolic compounds did not show differences with the levels of control plants (Figure 8A).

To determine the antioxidant role of phenolic compounds in *D. antarctica*, the scavenger capacity of the free radical DPPH was evaluated in ethanolic extracts obtained from treated and control plants. The scavenger capacity of the extract obtained in plants treated at 23°C, decreased by 35% on day 1 of treatment, but on day 7 it did not show differences with the control group (Figure 8B). Under the 35°C treatment, a slight increase of the antioxidant activity was observed on day 1, but no differences were observed at the end of the treatment on day 7.

## Discussion

High temperature is an important abiotic stress factor for plants that is not restricted to tropical areas and desert belts but also may play an important role in colder regions like the Antarctica (Convey, 2006). The Antarctic Peninsula has experienced rapid warming in recent decades. The warming may in turn cause critical overheating as the heat trapping nature can get fatal in situations with high solar irradiation input, combined to restricted transpiration and calm winds causing situations where the thermal high temperature thresholds can get exceeded.

High temperature induces metabolic imbalances that can cause an oxidative stress in plant cells. This effect results in the generation and accumulation of ROS, promoting oxidation of cellular components, hindering metabolic activities and affecting organelle integrity (Suzuki et al., 2012).

In the present study, was assessed with the hypothesis that *D. antarctica* has efficient antioxidant protection mechanisms in response to heat shock treatment, being able to maintain normal Physiology, photosynthetic performance, and redox state balance.

The results have shown that *D. antarctica* has a high maximum heat tolerance of 52°C (LT_50_). Traditionally, the method used to determine the TL_50_ is based on the measurement of electron leakage (EL). The method used in this work is based on the measurement of the maximum photosynthetic efficiency (Fv/Fm). Determining chlorophyll fluorescence is a good alternative for EL measurement because of their noninvasive and rapid performance, as well as their potential for estimating LT_50_ Fv/Fm. This method is sensible, non-invasive, early, fast and easy to use (Ehlert and Hincha, 2008). Previously, Reyes et al. (2003), reported that D. antarctica plants, grown in growth chambers at 4 and 13°C, presented a LT50 of 48.3°C when analyzing the membrane damage. This value is similar to the one determined in this work, although the methods used are totally different.

The value of LT_50_ determined is surprisingly high for a species that has lived in a rather cold environment. Due to global warming that affects the Antarctic region and the increasing heat waves it can be suggested that this species has the potential to tolerate warm temperatures.

Global warming has led to an increase in soil temperature, major abiotic stress that poses a difficult threat to plants. Photosynthesis is among the plant cell functions that are very sensitive to stress at high temperatures and often interfere before other cell functions deteriorate. The major targets for high-temperature stress are Photosystem II (PSII), and oxygen evolution complex (OEC), and ribulose-1,5-bisphosphate carboxylase/oxygenase (Rubisco) (Mathur et al., 2014). Previous studies have shown that the optimal photosynthetic temperature in *D. antarctica* is in a range between 13 and 19°C (Edwards and Smith, 1988; Xiong et al., 1999). Additionally, the carboxylase activity of Rubisco in *D. antarctica* has not shown differences at 5, 15, and 25°C (Sáez et al., 2017).

Related to the hypothesis of this work, the results show *D. antarctica* is able to tolerate a heat shock treatment of 23°C and 35°C. Elevated temperatures could also determine a loss in the content of photosynthetic pigments. It has been reported that due to the effect of heat, chlorophyllases and peroxidase are induced in plants (Rossi et al., 2017). However, *in D. antarctica*, the values of the chlorophyll a/b ratio remained in the range 1.8–2.5, which is characteristic of nonstressed plants (Yu et al., 2015). The null effect observed in the photosynthetic performance which means that the thylakoid membranes have maintained their functionality, without showing damage by lipid oxidation (lipoperoxidation), due to the accumulation of ROS. Photosynthetic activity at 35°C has not been previously reported in *D. antarctica* and shows its plasticity under unfavorable conditions such as those that are affecting the Antarctic region.

A key factor in tolerance to thermal stress is the ability of plants to rapidly control oxidative damage (Tognetti et al., 2012). The PSII complex is one of the most thermolabile components, mainly the D1 protein due to ROS attack (Wang et al., 2018). The results of this work show *D. antarctica* maintained a normal functioning of their photosynthetic machinery to both shock treatments. This was due at least in part to the efficient enzymatic antioxidant activity, which allowed it to control H_2_O_2_ levels, described as a potent inhibitor of photosynthesis in plants due de oxidation of the core components of PSII, like protein D1, and for the oxidation of other biomolecules like lipids, proteins among others (Foyer and Shigeoka, 2011). APX is one of the key enzymes to protect photosynthetic activity because it is located inside the chloroplasts that detoxify the H_2_O_2_ produced by SOD activity using ascorbate as a substrate (Roach and Krieger-Liszkay, 2014). Our results show an induction of APX activity at 23°C but not at 35°C. This is important, since it shows that this enzyme is activated only at certain stress thresholds.

Since there was no damage to *D. antarctica* in both treatments, it can be suggested that other mechanisms operate at 35°C instead of APX activity, which would act at 23°C. At 23°C during the first days of treatment, an increase in carotenoids levels is observed, a situation that does not occur at 35°C. Since the carotenoid level showed an increase in the first days of treatment at 23°C, its role may be related to the photoprotective properties. Its antioxidant activity (Nizar et al., 2015), alpha, beta carotenoids, and xanthophylls are agents stabilizers of the thylakoid membrane and reaction centers of the photosystems; these molecules are essential to prevent the leakage of ions from the tissues and act as antioxidants when they are free in the cell, protecting the membranes of the thylakoids from oxidation by the ROS attack (Latowski et al., 2011).

Another enzyme that showed significant activity at 23°C is POD. This enzyme has previously been reported to play an important role in the tolerance of *D. antarctica* to osmotic stress (Zamora et al., 2010) and UV-B radiation (Köhler et al., 2017). In addition to their antioxidant function, some apoplastic POD isoforms are related to the thickening of the cell wall and the polymerization of lignin (De Gara, 2004). Therefore, POD activity also contributes to maintaining low ROS levels, membrane integrity, and metabolic functionality under heat shock conditions. Gielwanoska et al., 2005, described that in *D. antarctica*, adverse environmental conditions stimulate growth, altering the lignification of the cell wall. In a field study subjecting *D. antarctica* plants to a passive heating treatment, it was shown that after the treatment the plants showed a higher fiber content, mainly cellulose, and higher sclerophylly index (Sáez et al., 2018). The catalase activity did not show significant changes in the heat shock treatment. This could be explained by its low affinity to H_2_O_2_ compared to peroxidases, which are present in most organelles (Zamora et al., 2010), and increases in CAT activity are also related to the photorespiration process in plants (Foyer et al., 2009).

As previously mentioned, the activity of the APX and POD enzymes was sufficient to maintain the oxidative state *in D. antarctica* in the treatment at 23°C. On the other hand, at 35°C these enzymes were not induced, and no damage was observed, that implies another control mechanism must be operating against ROS accumulation. In this sense, an increase in the content of phenolic compounds and an increase of the free radical trapping activity were shown. A severe heat shock of 35°C activates signaling cascades based on lipid and Ca^+2^ inputs that regulate gene expression (Saidi et al., 2011). Among these genes, those involved with certain secondary metabolites related to the acquisition of thermotolerance in plants will be activated (Bita and Gerats, 2013). The chemical structure of phenolic compounds allows them to act as free-radical species trappers through two mechanisms, by electron transfer (SET, Single Electron Transfer) or hydrogen transfer (HAT, Hydrogen Atom Transfer) (Ahmad et al., 2010). For this reason, the accumulation of phenolic compounds within chloroplasts contributes to the detoxification of ROS and free radicals, maintaining the functionality of the chloroplast (Zhao and Zou, 2002), which would explain the low APX activity in *D. antarctica* at 35°C. compared to treatment at 23°C.

The efficient control of ROS in plants subjected to heat shock resulted in the control of MDA levels and in the maintenance of photosynthesis under conditions of thermal stress (Chen et al., 2010). Chlorophyll stability has been proposed to be a key characteristic in maize varieties classified as thermotolerant (Shirdelmoghanloo et al., 2016). The definition of homochlorophilic plants has been proposed to those species that have the capacity to maintain the integrity of photosystems and the integrity of chlorophyll under conditions of oxidative imbalance, induced by heat shock (Tuba and Lichtenthaler, 2011). In this work, we demonstrated that *D. antarctica* achieved maintaining all essential physiological parameters despite heat shock treatment with similar features like thermotolerant species (Vierling, 1991; Nagarajan et al., 2010; Scafaro et al., 2010;.). Photosynthetic activity at 35°C has not been previously reported in *D. antarctica* and shows its plasticity under unfavorable conditions such as those that are affecting the Antarctic region.

The results allow us to suggest that tolerance to a shock of 35°C is strongly influenced by the accumulation of phenolic compounds. The ability of the plant to accumulate phenolic compounds is considered an evolutionary advantage, which allows it to tolerate adverse environmental conditions (Edreva et al., 2008). It has been reported that under abiotic stress conditions, an activation of the transcription of key enzymes of the phenylpropanoid pathway such as phenylalanine ammonia lyase (PAL), chalcone synthase (CHS), chalcone isomerase (CHI) and flavonol synthase (FLS) are induced (Sharma et al., 2019).

## Conclusion

The results shown in this work allow us to indicate that *D. antarctica* plants grown *in vitro* are capable of tolerating thermal shock conditions of 23 and 35°C. These temperatures are 8 and 20°C higher than the temperature at which the plants are grown in the growth chambers. The tolerance shown is related to efficient enzymatic and non-enzymatic antioxidant systems, which facilitate proper cell function by controlling ROS levels.

This response of *D. antarctica* to heat shock could be key to its success against the warming that affects the Antarctic continent.

## Data Availability Statement

The raw data supporting the conclusions of this article will be made available by the authors, without undue reservation.

## Author Contributions

RC-A designed and conducted all experiments in the laboratory at University of Santiago and wrote the first draft of the manuscript. MP, RC, and HK collaborated in some analysis. MP, RC, and GZ collected samples in Antarctica. All authors contributed equally to the discussion and revision of the final version of the manuscript.

## Conflict of Interest

The authors declare that the research was conducted in the absence of any commercial or financial relationships that could be construed as a potential conflict of interest.
